# 电磁导航支气管镜在肺外周结节诊断中的应用

**DOI:** 10.3779/j.issn.1009-3419.2020.102.05

**Published:** 2020-06-20

**Authors:** 孟华 薛, 建 汪, 勇 韩, 以芳 朱, 娜 张, 晋波 赵, 小飞 李

**Affiliations:** 710038 西安，空军军医大学第二附属医院（唐都医院）胸外科 Department of Thoracic Surgery, the Second Hospital (Tang Du Hospital) Air Force Military Medical University, Xian 710038, China

**Keywords:** 电磁导航支气管镜, 肺外周病变, 活检, Electromagnetic navigation bronchoscopy, Peripheral lung lesions, Biopsy

## Abstract

**背景与目的:**

电磁导航支气管镜是一种能够进行肺部病变精准定位的工具，在肺部病变诊断中具有重要价值，但由于价格昂贵，在我国应用尚未普及，急需总结其在临床中的应用，尤其是肺部结节诊断中的经验。

**方法:**

回顾性分析我科2017年7月-2018年12月诊断为肺外周病变，且行电磁导航支气管镜检查患者的临床资料，总结其应用经验。

**结果:**

18例患者（男性10例，女性8例），共21处病变，11例诊断明确，8例诊断为肺腺癌，1例诊断为肺结核，2例诊断为小细胞肺癌，诊断阳性率为61.1%，敏感性为73.3%，诊断阳性率与病变大小相关， > 2 cm病变诊断阳性率为100.0%（*P*=0.04）。

**结论:**

电磁导航支气管镜在临床中安全有效，对于病变 > 2 cm的肺外周病变诊断阳性率较高，有着广阔的临床应用前景。

## Abstract (en)

**Background and objective:**

Electromagnetic navigation bronchoscopy is a tool that can accurately navigation peripheral lung lesions. Because of electromagnetic navigation bronchoscopy (ENB) is too expensive, it has not been widely used in China. It is urgent for us to summarize experience in clinical application, especially in the diagnosis of pulmonary nodules.

**Methods:**

The clinical data of patients with pulmonary peripheral lesions (PPLs) in our department undergoing ENB biopsy between July 2017 and December 2018 were retrospectively analyzed.

**Results:**

There were 18 patients with 21 PPLs (10 males and 8 females). Among them, 11 patients got the final pathological diagnosis, 8 cases were diagnosed with adenocarcinoma lung cancer, 1 case was diagnosed with tuberculosis and 2 cases were diagnosed with small cell lung cancer. The positive rate of diagnosis was 61.1%. The sensitivity was 73.3%. The positive diagnosis rate is related to the size of the lesion, the positive diagnosis rate for lesions > 2 cm is 100.0% (*P*=0.04).

**Conclusion:**

Electromagnetic navigation bronchoscope is safe and effective in clinic. It has a high positive rate for the diagnosis of peripheral lung lesions larger than 2 cm, ENB has broad clinical application prospects.

肺癌是我国发病率和死亡率第一的恶性肿瘤，晚期肺癌的生存率为20%，随着低剂量薄层计算机断层扫描（computed tomography, CT）的广泛推广，肺癌的死亡率降低了20%，越来越多的患者以肺外周病变（peripheral pulmonary lesions, PPLs）就诊，为临床诊断提出了新的挑战。微创诊断PPLs的方法包括支气管镜透支气管壁肺活检术（transbronchial lung biopsy, TBLB）及CT引导经皮肺穿刺术。TBLB阳性率仅为14%^[1]^，CT引导下经皮肺穿刺活检术阳性率高达90%，而气胸的发生率为24%，病变 < 2 cm的肺外周结节经皮肺穿气胸发生率可高达30%，并且经皮肺穿刺活检术有肿瘤转移种植的可能性^[2]^，目前临床急需一种安全、有效诊断PPLs的方法。

电磁导航支气管镜（electromagnetic navigation bronchoscopy, ENB）是一项新出现的临床技术，于1995年由以色列人Pinchas Gilboa发明，1998年约翰霍普金斯放射科的Solomon第一次提出电磁导航有助于支气管镜定位，2001年电磁导航系统与CT三维成像结合，并引入可调节方向的导管，ENB在动物实验中取得良好的效果，2003年开展第一例人体检查，随着大量临床研究的开展及诊断阳性率的提高^[3]^，ENB被广泛应用于PPLs活检，并在近两年来逐步开始应用于PPLs手术定位和微波消融治疗。

目前ENB在我国仅有少数单位开展，现回顾我科自2017年7月-2018年12月18例ENB检查临床病例资料，总结ENB在PPLs诊断中的应用经验，为进一步治疗和手术提供依据，探索ENB在胸外科领域的应用。

## 1 材料与方法

### 1.1 一般资料

回顾性分析空军军医大学附属第二医院（唐都医院）胸外科于2017年7月-2018年12月行ENB检查的18例患者，21处病变，年龄53岁-80岁，平均年龄66.7岁，男性10例，其中8例有吸烟史，2例无吸烟史，女性8例，均无吸烟史，病变位于右肺上叶6处，右肺中叶3处，右肺下叶4处，左肺上叶8处，其中3例患者有2处病变（图 1），病变大小为11 mm-35 mm，中位大小17 mm，其中15处病变小于20 mm，6处病变大于20 mm。病变距胸膜距离5 mm-53 mm，中位距离16 mm，15处病变无支气管通气症，6处病变有支气管通气症。

**1:**
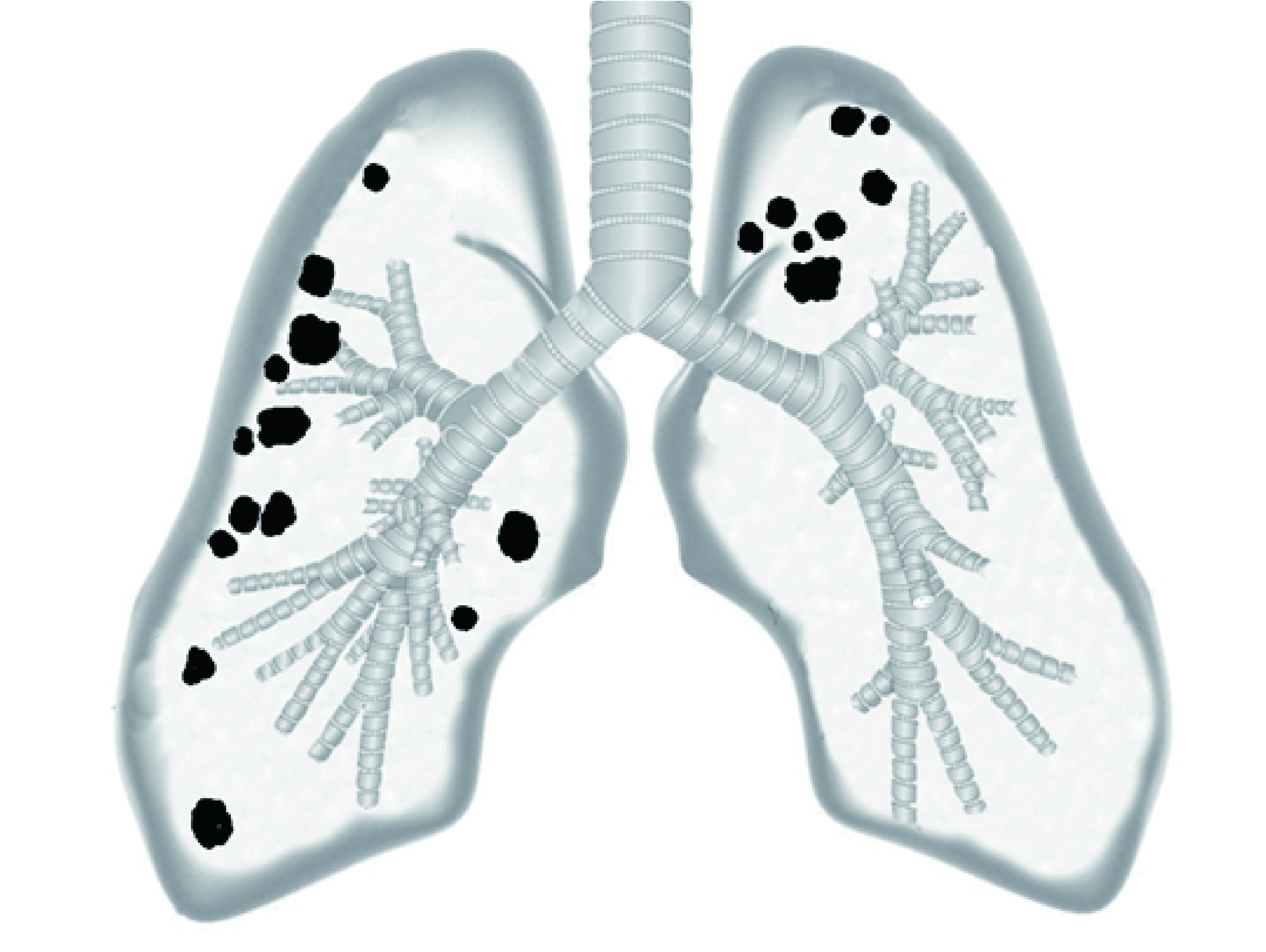
21处PPLs病变分布图，右肺上叶6处，右肺中叶3处，右肺下叶4处，左肺上叶8处。 PPLs map of 21 nodules, 6 nodules in right upper lobe, 3 nodules in right middle lobe, 4 nodules in right lower lobe, 8 nodules in left upper lobe. PPLs: peripheral pulmonary lesions.pulmonary lesions

### 1.2 纳入与排除标准

纳入标准：①完善术前检查（血常规、出凝血检查、心电图、心脏彩超、肺功能），无检查禁忌症，能耐受ENB检查及全麻；②检查前胸部CT提示病变位于肺外周，常规气管镜检查无法取到活检；③检查前无明确病理诊断；④征求家属知情同意并签署知情同意书。排除标准：①未成年人或者体格瘦小者，6级支气管直径 < 2 mm，无法插入引导鞘管；②一般情况差、体质衰弱不能耐受支气管镜检查者；③有精神不正常，无法配合CT检查者；④急性心血管疾病者，如不稳定性心绞痛、心肌梗塞、严重心律失常、严重心功能不全、主动脉瘤等；⑤麻醉药物过敏者，无法用其他药物代替者；⑥严重出血倾向及凝血机制障碍者；⑦无法获得知情同意者。

### 1.3 ENB操作方法

#### 1.3.1 ENB原理

美敦力公司Super Dimension电磁导航系统是基于电磁导航系统、虚拟支气管镜和三维CT相结合的一种新技术，利用磁场的原理，将一块可以产生微磁场的定位板（Location Board）置于患者身下，在患者体表放置三联体传感器（Patient Sensors）做基本定位（导航卫星），利用胸部薄层CT扫描重建虚拟3D支气管树（地图），设计经支气管到PPLs的路径，将可见感应到微磁场的定位导向管（locatable guide, LG）（导航仪）按照术前规划好的路径，通过推进和调节导向管方向到达病灶。

#### 1.3.2 操作流程

① 术前规划路径：胸部薄层CT扫描，扫描层厚为1 mm-1.25 mm，层间距为0.8 mm-1.0 mm，扫描层数50层-690层，图像分辨率512×512，并以DICOM格式并刻录光盘，用磁导航系统术前计划电脑读取光盘，重建虚拟支气管，标记隆突，右主支气管、右肺中叶、右肺下叶、左主支气管、左肺下叶五个注册点，设定目标病灶，寻找到达目标病灶的路径并做标记。②电磁导航术前准备：将已规划好路径的数据复制到电磁导航系统主机，在患者身下置入定位板并将其与系统连接，将三联体传感器分别置于患者胸骨角及双侧第8肋骨与腋中线连线处，确定其位于磁场中，按照电磁导航扩展工作通道（extended working channel, EWC）长度标记活检钳。③ENB活检：采取喉罩全麻，置入支气管镜（BF-1T260, Olympus, Tokyo, Japan），将电磁导航EWC及LG锁定，经支气管镜活检通道置入，按照提示完成注册，注册结束后，按照术前规划好的路径，不断调节导航探头的方向，将EWC及LG送到病灶附近，保留EWC，拔出LG至气管后再次将LG送至病灶，确定位置无变化，拔出LG并将有标记的活检钳送入EWC活检，活检数次后将LG再次送入确定位置，反复数次，直到取到满意组织即可。

### 1.4 结果判定标准

经病理学家明确得出诊断，如肺癌，肺结核等，定义为明确诊断；对于病理学家仅仅描述为炎性渗出、坏死及纤维组织增生等情况定义为不明确诊断。

### 1.5 统计学方法

采用SPSS 24.0统计学软件对数据进行分析，计数资料比较采用*χ*^2^检验，*P* < 0.05为差异有统计学意义。

## 2 结果

18例患者，其中V6导航系统检查17例，V7导航系统检查1例，V6导航系统设计路径用时10 min-48 min，所有病例均在V7导航系统中重新设计路径，设计路径时间为3 min-10 min，导航到达病灶时间（包含注册、导航、到达病变活检）为25 min-80 min。18例电磁导航支气管活检明确病理诊断11例，其中诊断为腺癌8例，5例行基因检测为阳性，口服靶向药物治疗，3例基因检测为阴性，诊断为结核1例，诊断为小细胞肺癌2例，未明确诊断7例，其中3例拒绝进一步检查，4例行胸腔镜楔形切除术，术后病理诊断为腺癌（见表 1）。诊断阳性率为61.1%，敏感性为73.3%，病变 < 2 cm有15处，其中8处明确诊断，诊断阳性率为53.3%，病变 > 2 cm有6处，其中6例明确诊断，诊断阳性率为100%（*P*=0.04），无支气管通气症病变15处，其中9例明确诊断，诊断阳性率为60%，有支气管通气症病变6处，其中5例明确诊断，诊断阳性率为83.3%（*P*=0.306）（表 2），活检中未发生气胸及出血并发症。

**1:** 患者一般情况 Clinical characteristics of patients

Characteristics		Data
Gender	Male	10 (55.6%)
	Female	8 (44.4%)
Smoking history	Smoking	8 (44.4%)
	Non smoking	10 (55.6%)
Pathologic	Adenocarcinoma	8 (44.4%)
	SCLC	2 (11.1%)
	Tuberculosis	1 (5.6%)
	Negative	7 (38.9%)
Tumor location	LUL	8 (38.1%)
	RUL	6 (28.6%)
	RML	3 (14.3%)
	RLL	4 (19.0%)
Size of lesion	≤2 cm	15 (71.4%)
	> 2 cm	6 (28.6%)
Air bronchograms of lesion	Positive	5 (23.8%)
	Negative	16 (76.2%)
SCLC: small cell lung cancer; LUL: left upper lobe; RUL: right upper lobe; RML: right middle lobe; RLL: right lower lobe.		

**2:** 病变大小以及有无支气管通气症与诊断的关系 Comparisons of size and air bronchograms for the diagnosis of lesions

	Positive	Negative	*P*
Size of lesion ≤2 cm	8 (53.3%)	7 (46.7%)	0.04
Size of lesion > 2 cm	6 (100.0%)	0 (0.0%)	
Air bronchograms (+)	5 (83.3%)	1 (16.7%)	0.306
Air bronchograms (-)	9 (60.0%)	6 (40.0%)	

## 3 讨论

### 3.1 ENB在临床诊断中的应用价值

越来越多的研究显示，ENB对于肺外周疾病的诊断具有重要的价值。Rivera等关于ENB活检的前瞻性研究显示诊断阳性率为68%，而回顾性研究的诊断阳性率为71%^[4]^，比我们研究的阳性率略高。在我们的研究中有1例患者发现右肺病变3年，2017年左肺上叶出现磨玻璃样病变，右侧病变大小无变化，对右肺上叶及左肺上叶病变均行ENB检查，右肺上叶准确到达病变并活检，左肺上叶病变导航时，由于支气管管腔狭窄，用活检钳扩通支气管后，导航扩展通道仍无法进入，在距病变1.6 cm处将已标记活检钳往后延伸标记1.6 cm后取活检，患者两处活检病理均考虑肺组织慢性炎伴炎性渗出及纤维组织增生，未进一步检查出院，归类到未明确诊断组，因此导致活检阳性率略低。Gex等^[5]^对1, 033例PPLs采用亚组分析和*meta*回归分析研究显示，ENB诊断阳性率介于55.7%-87.5%之间，敏感性为71.1%，Zhang等^[6]^对1, 106例PPLs患者做*meta*分析显示诊断敏感性高达100%，这与我们研究的诊断阳性率（61.1%）及敏感性（73.3%）相似。但Ost等^[7]^报道一项关于581例ENB的前瞻性研究得出了相反的结论，单独ENB活检阳性率低于单独支气管镜检查，仅38.5%的患者经电磁导航系统确诊，由于此项研究在选择病例、麻醉方法、取样方法以及麻醉方法中存在差异，同样在我们的研究中也存在病例选择的差异，我们选用常规支气管镜未确诊的肺外周病变患者，使导致诊断阳性率降低。另外Ost等^[7]^认为ENB活检率低与在同一部位活检有关，导致活检“全有”或“全无”，而我们在行常规支气管镜活检中，两次活检钳取同一部位的概率极低，而磁导航系统在升级到V7系统后，已将此做优化，可以对活检部位标记，以免重复在同一个地方活检造成“全有”或“全无”，使活检阳性率提高到96.8%^[8]^。

Sun等^[9]^认为ENB的诊断率与性别、年龄、病灶大小、病灶位置、气道与结节的关系、结节与肺胸膜的距离无关，Brownback等^[10]^对55例PPLs研究显示有无支气管通气征对诊断阳性率无影响（*P*=0.35），病灶直径 > 3 cm的病变与直径 < 3 cm的病变二者诊断阳性率无显著增加（*P*=0.22）。我们的研究也显示支气管通气症对明确诊断无影响（*P*=0.306），但在我们的研究中发现，病变越大ENB活检的阳性率越高，病变 > 2 cm比病变 < 2 cm诊断阳性率显著增加（*P*=0.04）。

### 3.2 如何提高ENB诊断阳性率

单独应用ENB检查阳性率介于67%-84%^[3]^，Eberhardt等对118例经ENB和径向超声探头（radial endobronchial ultrasound, r-EBUS）检查的随机性研究表明，ENB的诊断阳性率为59%，而r-EBUS的阳性率为69%，而联合应用二者可使诊断的阳性率提高到88%，高于单独使用（*P*=0.02）^[11]^。此外Lamprecht等报道ENB联合细胞学快速现场评估（rapid on-site cytopathologic evaluation, ROSE）的诊断敏感性为84.6%，特异性为100%^[12]^。因此，ENB检查时如果能联合使用r-EBUS、ROSE，可提高诊断阳性率，这也是我们后期研究中需要弥补的地方。另外，Samuel等^[13]^报道，相同条件下不同的活检方式诊断阳性率不同，活检钳活检的诊断率为75%，细针穿刺活检诊断率为73%，而细胞刷检和支气管肺泡灌洗（bronchoalveolar lavage, BAL）的诊断率分别为41%和为30%，由于刷检和灌洗的易用性及安全性，尤其适用于感染患者，虽然诊断阳性率低，但仍在临床中广泛应用。黄海涛等^[14]^将ENB与r-EBUS相结合，同时使用细针穿刺、细胞刷检、活检，诊断阳性率达90%。因此在行ENB检查时不仅仅是只做活检，还应当联合刷检、灌洗，尤其是穿刺活检（trans-parenchymal nodule access, TPNA），以提高诊断阳性率。

### 3.3 ENB在临床中的其他应用

PPLs术中定位一直是胸外科的难题，目前常采用的方法是CT引导下经皮肺穿放置弹簧圈或者注入凝胶来标记病变，但是气胸的发生风险高达30%^[15]^，而且医护人员要遭受电离辐射，随着ENB——“精准到达病变”——被越来越多的临床医生认可，而且ENB无放射线暴露的危险，因此术前利用ENB标记PPLs逐步在临床中应用。Krimsky等^[16]^对21例经ENB染色标记胸膜的患者做回顾性研究表明，81%的病例在手术时脏胸膜可染色标记，并且经靛蓝胭脂的病变3 d后仍可以看到染色剂。因此对于无法在手术室行ENB检查的单位推荐行靛蓝胭脂染色。Luo等^[17]^改良了此方法，通过对30例患者的PPLs注入生物胶、凝血酶及亚甲蓝染色，术中可更明显触摸到结节，提高术中定位准确性。但是亚甲蓝染色对于一些长期抽烟且吸烟量较大的患者可能出现染色不明显，随着胸腔镜荧光内镜的广泛应用，可以通过ENB注入荧光染色剂，使病变部位在术中更直观。

立体定位放疗对于无法耐受手术的PPLs患者是一个较理想的治疗方法。对于病变较小而无法准确定位者，可以选择ENB标记定位。Hagmeyer^[18]^报道了6例经ENB放置放疗基准定位器，其中3例患者由于咳嗽，14 d后出现定位器移位，再次定位后均治疗成功，并且无放疗相关并发症。

ENB检查对肺移植患者也一样适用，Panchabhai^[19]^报道了10例肺移植术后新发PPLs患者行ENB检查，并联合r-EBUS及ROSE，其中7例诊断为感染，2例诊断为肺癌，1例阴性，因此ENB对于肺移植术后的结节不仅安全，而且有着较高的诊断率，是否能应用于肺叶切除术后患者的诊断和治疗目前尚无报道，后期需要我们持续研究，为术后PPLs患者诊断和无创治疗提供依据。

### 3.4 ENB检查的缺点

ENB规划路径需要参照薄层CT扫描，而在规划路径后如果病变有变化，无法“更新路径”，并且在活检中无法用实时监测判断是否到达病变，这两种情况都会导致活检失败，因此在胸部CT扫描后应尽快检查以减少误诊。此外ENB检查费用较高，目前仅耗材费用需要1.3万元，与腔镜肺切除术费用相当，对有些中低收入家庭来说是一笔不小的支出，也严重制约了其进一步的临床应用。随着技术的提高以及医保政策的调整，这些缺点会逐渐得到克服。

总之，ENB对PPLs活检安全有效，对病变 > 2 cm的诊断阳性率较高，联合r-EBUS、ROSE以及多种活检方式可进一步提高活检阳性率，随着电磁导航技术的不断完善提高，可为更多的患者提供精准检查及治疗。
